# Reconstruction of the Evolutionary Dynamics of the A(H1N1)pdm09 Influenza Virus in Italy during the Pandemic and Post-Pandemic Phases

**DOI:** 10.1371/journal.pone.0047517

**Published:** 2012-11-09

**Authors:** Gianguglielmo Zehender, Elena Pariani, Antonio Piralla, Alessia Lai, Elena Gabanelli, Alberto Ranghiero, Erika Ebranati, Antonella Amendola, Giulia Campanini, Francesca Rovida, Massimo Ciccozzi, Massimo Galli, Fausto Baldanti, Alessandro Remo Zanetti

**Affiliations:** 1 Dipartimento di Scienze Cliniche e Biomediche “Luigi Sacco”, Sezione di Malattie Infettive, Università degli Studi di Milano, Milan, Italy; 2 Dipartimento di Scienze Biomediche per la Salute, Università degli Studi di Milano, Milan, Italy; 3 Struttura Semplice Virologia Molecolare, Struttura Complessa Virologia e Microbiologia, Fondazione Istituto Ricovero e Cura a Carattere Scientifico Policlinico San Matteo, Pavia, Italy; 4 Istituto Superiore di Sanità, Dipartimento Malattie Infettive, Parassitarie ed Immunomediate, Rome, Italy; The University of Hong Kong, China

## Abstract

The aim of this study was to reconstruct the evolutionary dynamics of the A(H1N1)pdm09 influenza virus in Italy during two epidemic seasons (2009/2010 and 2010/2011) in the light of the forces driving the evolution of the virus. Nearly six thousands respiratory specimens were collected from patients with influenza-like illness within the framework of the Italian Influenza Surveillance Network, and the A(H1N1)pdm09 hemagglutinin (HA) gene was amplified and directly sequenced from 227 of these. Phylodynamic and phylogeographical analyses were made using a Bayesian Markov Chain Monte Carlo method, and codon-specific positive selection acting on the HA coding sequence was evaluated. The global and local phylogenetic analyses showed that all of the Italian sequences sampled in the post-pandemic (2010/2011) season grouped into at least four highly significant Italian clades, whereas those of the pandemic season (2009/2010) were interspersed with isolates from other countries at the tree root. The time of the most recent common ancestor of the strains circulating in the pandemic season in Italy was estimated to be between the spring and summer of 2009, whereas the Italian clades of the post-pandemic season originated in the spring of 2010 and showed radiation in the summer/autumn of the same year; this was confirmed by a Bayesian skyline plot showing the biphasic growth of the effective number of infections. The local phylogeography analysis showed that the first season of infection originated in Northern Italian localities with high density populations, whereas the second involved less densely populated localities, in line with a gravity-like model of geographical dispersion. Two HA sites, codons 97 and 222, were under positive selection. In conclusion, the A(H1N1)pdm09 virus was introduced into Italy in the spring of 2009 by means of multiple importations. This was followed by repeated founder effects in the post-pandemic period that originated specific Italian clades.

## Introduction

In March 2009, a novel swine-derived A(H1N1) influenza virus – A(H1N1)pdm09 – emerged in Mexico and started spreading across the globe, prompting the World Health Organisation (WHO) to raise the level of influenza pandemic alert to phase 6 (WHO – available at: http://www.who.int/csr/disease/swineflu/en/). Despite the rapidity with which the virus reached a large number of countries in the world, its transmission was initially sustained only in a subset of countries, particularly the USA and temperate countries in the southern hemisphere in which winter influenza transmission was ongoing and a full A(H1N1)pdm09 influenza epidemic was observed. The pandemic strain quickly became the predominant circulating influenza virus and replaced seasonal strains in most countries.

There was considerable heterogeneity in the pattern of A(H1N1)pdm09 spread in Europe. The UK experienced a substantial first wave of transmission in the early summer, followed by a second in the autumn, whereas most European countries (including Italy) experienced only limited transmission before the summer and a single wave in the autumn of 2009 [1]. A second epidemic wave was recorded during the post-pandemic period (November 2010–March 2011) during which the influenza A(H1N1)pdm09 virus was responsible for the majority of infections.

A(H1N1)pdm09 is a novel reassortant virus containing genes from the North American triple reassortant swine viruses and neuraminidase (NA) and matrix (M) genes derived from Eurasian swine viruses. It had probably been circulating undetected among swine during the previous decade, but only recently emerged among humans [2], [3].

The emergence and subsequent rapid global spread of this influenza virus provided a unique opportunity to observe the evolutionary population dynamics of the first influenza pandemic virus after forty years, particularly in regions where virological surveillance is comprehensive, closely matched to the well-defined chronology of epidemic waves, and related disease surveillance (available at the Italian Influnet website: http://www.iss.it/iflu/). The molecular characterisation of the A(H1N1)pdm09 pandemic revealed seven globally distributed main clades [4].

The aim of this study was to reconstruct the evolutionary dynamics of the A(H1N1)pdm09 influenza virus in Italy during two epidemic seasons (2009/2010 and 2010/2011) in the light of the forces driving viral evolution.

## Patients and Methods

### Ethics statement

According to the Regional Surveillance and Preparedness Plan (DGR IX/1046, 22 Dec. 2010 and DGR 5988, 30 Jun 2011), diagnostic and clinical management of patients admitted at hospitals in the Lombardy Region with severe and moderate ILI included prospective influenza A detection, subtyping and sequencing. These activities were centralized at the two regional reference laboratories (S.S. Virologia Molecolare, Fondazione IRCCS Policlinico San Matteo, Pavia, and Dipartimento di Scienze Biomediche per la Salute, Università degli Studi di Milano, Milan). Mild respiratory infections were collected by sentinel practitioners and anonymously analyzed at the reference laboratory in Milan, in the frame of the National Surveillance Plan (Influnet). Data were analyzed anonymously according to a Regional Surveillance and Preparedness Plan. Mild ILI were collected and analyzed within the National Surveillance Plan (Influnet), following approval by the Ethic Commitee of Fondazione IRCCS Policlinico San Matteo, Pavia.

### Dataset and patients

Within the framework of the Italian Influenza Surveillance Network, nasal swabs (NS) or broncho-alveolar lavages (BAL) were collected from outpatients with the symptoms of influenza-like illness (ILI) and hospitalised patients suffering from severe respiratory syndromes.

During the pandemic (May 2009–April 2010) and post-pandemic period (May 2010–April 2011), 5,844 respiratory specimens were collected in Lombardy (Northern Italy) and sent to the regional reference laboratories (S.S. Virologia Molecolare, Fondazione IRCCS Policlinico San Matteo, Pavia, and Dipartimento di Scienze Biomediche per la Salute, Università degli Studi di Milano, Milan) for the virological diagnosis of A(H1N1)pdm09 infection.

A dataset was constructed that included 227 HA gene sequences (835 nucleotides in length, positions 121–954) obtained from as many A(H1N1)pdm09-positive patients, whose characteristics are shown in Table S1.

### A(H1N1)pdm09 detection and HA sequencing

Total RNA was extracted from the respiratory samples using the Nuclisens® easyMAG™ automated extraction kit (BioMerieux, Lyon, France), and a virological diagnosis of A(H1N1)pdm09 infection was made by means of a real-time reverse-transcriptase polymerase chain reaction (RT-PCR) assay [5]. The A(H1N1)pdm09 HA gene was amplified directly from the clinical specimens using a SuperScriptIII^TM^ One-step RT-PCR amplification kit (Invitrogen, Carlsbad, USA) and an RT-PCR assay specific for a 995 bp fragment (nt. positions 64–1,058) in the HA1 domain [6]. The PCR products were purified using a Microcon-100 microconcentrator in accordance with the manufacturer's instructions (Millipore, Bedford, MA, USA), and the purified products were sequenced using a BigDye Terminator Cycle-Sequencing kit (Applied Biosystems, Foster City, USA) and ABI Prism 3100 DNA sequencer (Applied Biosystems, Foster City, USA).

The sequences were deposited with GenBank, National Center for Biotechnology Information (NCBI) (http://www.ncbi.nlm.nih.gov). Their accession numbers: GQ149765, GQ246478, GQ259997–GQ260001, GQ330653–GQ330655, GQ374889, GQ374891, GQ374892, GQ422377–GQ422381, GQ387381–GQ387384, GQ258717, GQ166222, GQ259996, GU451262–GU451280, GU459082–GU459139, JF801855–JF801909, JN017095–JN017181.

### Phylogenetic analysis

The sequences were aligned using CLUSTALW (integrated within the Bio-Edit sequence editor by Tom Hall, 2001; http://www.mbio.ncsu.edu/BioEdit/bioedit.html). The best-fitting nucleotide substitution model was estimated by means of JModeltest [7], and selected an HKY model [8] with gamma-distributed rates among sites.

The phylogenetic tree, model parameters, evolutionary rates and population growth were co-estimated using a Bayesian Markov Chain Monte Carlo (MCMC) method implemented in the BEAST v.1.54 package [9]. Statistical support for specific clades was obtained by calculating the posterior probability of each monophyletic clade. As coalescent priors, we compared four simple parametric demographic models (constant population size, and exponential, expansion and logistic population growth) and a piecewise-constant model, the Bayesian skyline plot (BSP) under both a strict and a relaxed (uncorrelated log-normal) clock [10].

Two independent MCMC chains were run for 100 million generations with sampling every 10,000^th^ generation, and were combined using the LogCombiner 1.54 included in the BEAST package. Convergence was assessed on the basis of the effective sampling size (ESS) after a 10% burn-in using Tracer software version 1.5 (http://tree.bio.ed.ac.uk/software/tracer/). Only ESS's of ≥200 were accepted. Uncertainty in the estimates was indicated by 95% highest posterior density (95% HPD) intervals, and the best-fitting models were selected using a Bayes factor (BF with using marginal likelihoods) implemented in BEAST [11]. In accordance with Kass and Raftery [12], the strength of the evidence against H_0_ was defined as 2lnBF<2 =  none; 2–6 =  weak; 6–10 =  strong; and >10 =  very strong. A negative 2lnBF indicates evidence in favour of H_0_. Only values of ≥6 were considered significant. The trees were summarised in a target tree using the Tree Annotator program included in the BEAST package, choosing the tree with the maximum product of posterior probabilities (maximum clade credibility) after a 10% burn-in.

The basic reproductive number (R_0_), indicating the mean number of secondary cases generated by a single primary case, was estimated on the isolates sampled during the pandemic period. It was calculated on the basis of the exponential growth rate (r) using the equation R_0_  =  rD+1, where D is the average duration of infectiousness [13], assuming a generation time similar to that of other pandemic viruses [14], [15]. The doubling time of the epidemic was given by the relation λ =  ln(2)/r [16].

### Bayesian phylogeography

The spatial reconstruction was obtained by means of the same Bayesian framework using a continuous time Markov Chain (CTMC) implemented in BEAST [17] over discrete sampling locations, and applying a Bayesian stochastic search variable selection (BSSVS) model, which allows the diffusion rates to be zero with a positive prior probability. Comparison of the posterior and prior probabilities of the individual rates being zero provided a formal BF to test the significance of the linkages between locations. Rates yielding a BF of >6 were considered well supported and formed the migration pathway. The 227 Italian isolates were grouped into 14 localities corresponding to different regions of northern Italy: Milan (MI), Monza (MB), Bergamo (BG), Brescia (BS), Cremona (CR), Varese (VA), Como (CO), Lecco (LC), Mantua (MN), Pavia (PV), Parma (PR), Rimini (RN), Savona (SV), and Palermo (PA). The maximum clade credibility (MCC) tree was selected from the posterior tree distribution using the TreeAnnotator program after a 10% burn in. The final trees were manipulated in FigTree v.1.3 for display purposes.

### Selection pressure analysis

The d_N_/d_S_ ratio (ω) was estimated using the maximum likelihood (ML) approach under a global single-ratio model implemented in the HyPhy program [18]. In particular, the global model (which assumes a single selective pressure for all branches) was compared with the local model (which allows selective pressure to change along every branch) using the likelihood ratio test (LRT). The second model was not better than the first in any of the patients.

Site-specific positive and negative selections were estimated using three different algorithms: single likelihood ancestor counting (SLAC), derived from the Suzuki-Gojobori approach [19]; fixed-effects likelihood (FEL), which fits an ω ratio to every site and uses the likelihood ratio to test whether d_N_ ≠ d_S_; and random effect likelihood (REL), a variant of the Nielsen-Yang approach [20], which assumes the existence of a discrete distribution of rates across sites, and allows both d_S_ and d_N_ to vary independently site-by-site. The three methods were described in more detail elsewhere [18].

Finally, in order to investigate whether the sampled sequences have been subjected to selective pressure at population level (i.e. along internal branches), an internal fixed effects likelihood (IFEL) method [21] was also used.

In order to select the sites under selective pressure, we assumed a p value of ≤0.1 or a posterior probability of ≥0.9. The likelihood ratio test (LRT) was used to compare the performances of the M0 (one-ratio), M1 (nearly-neutral) and M2 (selection) models. Hyphy software was used for all of the analyses, some of which were made using the web-based Datamonkey interface (http://www.datamonkey.org/) [22].

## Results

### Global phylogenetic analysis and population dynamics of the Italian A(H1N1)pdm09 epidemics

**Figure 1:**
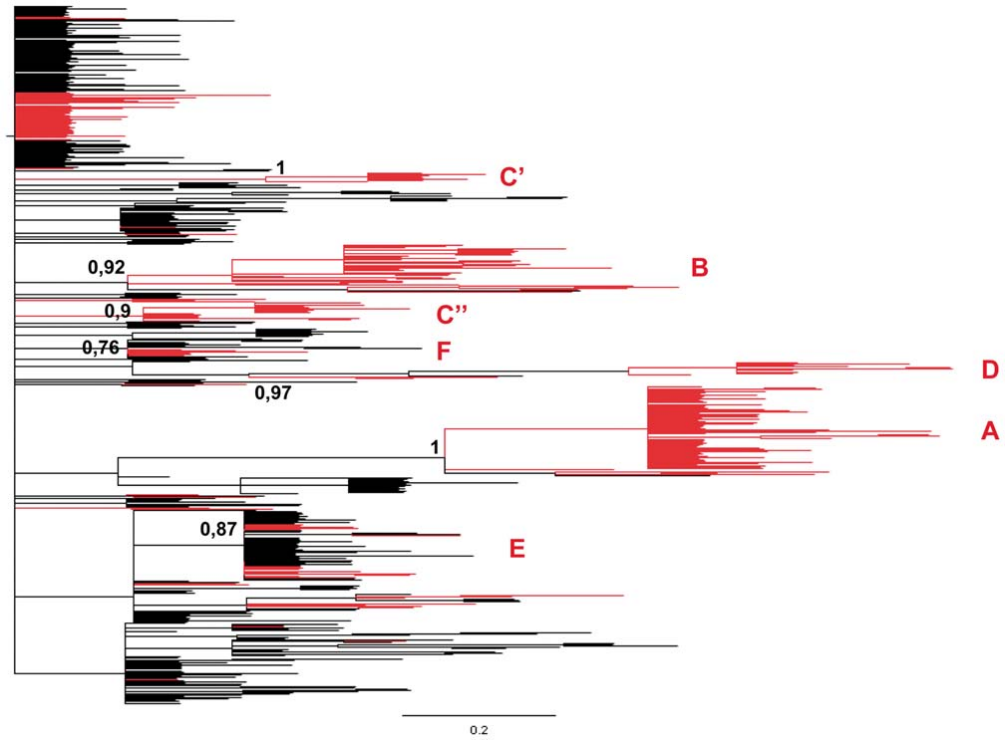
Bayesian MCMC phylogenetic tree of 561 influenza A(H1N1)pdm09 HA sequences. The 227 Italian isolates newly characterised in this study are highlighted in red. The specific Italian clades (A–D) including more than two isolates and having a posterior probability of ≥0.9 are shown, as are two main clades including several Italian and non-Italian strains (E and F). The letters indicate the position of the identified clusters, and the scale bar the number of substitutions.

The maximum likelihood and Bayesian analyses of the global data set of 561 A(H1N1)pdm09 isolates (227 from Italy and 334 from all over the world) showed that the Italian isolates clustered into five significant groups (pp>0.9). The clusters included a total of 136 isolates, representing 59.9% of the Italian isolates and 100% of those of the post-pandemic season (2010/2011), whereas the isolates of the pandemic season (2009/2010) were interspersed with sequences from other countries (Figure 1).

In order to reconstruct the population dynamics of the A(H1N1)pdm09 epidemics in Italy, we separately analysed the Italian isolates with a known month of isolation, and estimated the evolutionary rate.

### Evolutionary rate estimates

A strict and a relaxed (log-normal) molecular clock model were implemented under the less stringent Bayesian skyline plot demographic model. The marginal likelihood comparison showed that the relaxed clock did not fit the data significantly better than the strict clock (2lnBF = 9.85). Moreover, the lower 95% HPD limit of the coefficient of variation and the evolutionary rate standard deviation estimates were always very small (respectively 1.01×10^−5^ and 1.3×10^−4^), thus indicating that the evolutionary rate varied only slightly over the branches of the tree. For these reasons, the strict clock model was selected for all of the subsequent analyses.

Under this condition, we estimated a mean evolutionary rate of 4.15×10^−4^ subs/site/month (95% HPD: 2.9–5.3×10^−4^). The evolutionary rates of the 1^st^ + 2^nd^ codon positions (μ_1_, mean relative substitution rate 0.75, 95% HPD 0.64–0.87) were significantly lower than that of the 3^rd^ codon position (μ_2_, mean relative substitution rate 1.5, 95% HPD: 1.3–1.7).

### Temporal dynamics

**Figure 2:**
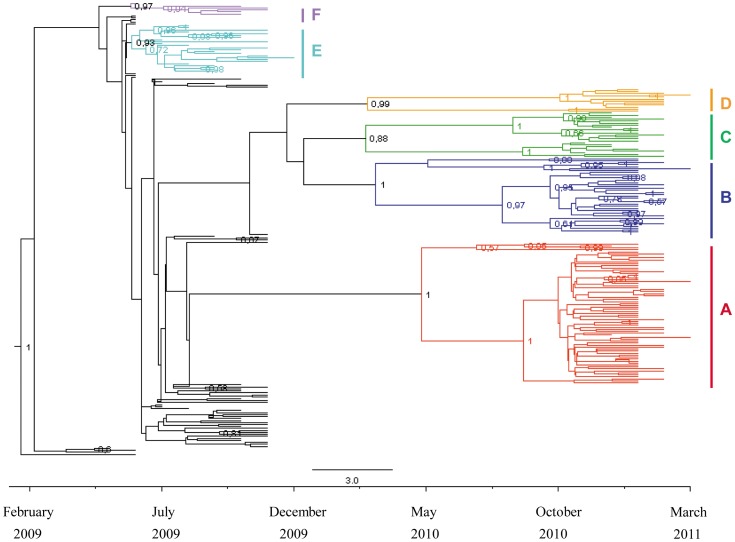
Time-scaled MCC tree of the 227 Italian influenza A(H1N1)pdm09 HA sequences. The main significant clades described in the text are highlighted in different colours. The numbers on the internal nodes indicate posterior probabilities. The bar at the bottom of the tree represents the calendar months between the tMRCA of the tree root and the most recent samples (March 2011).

The Bayesian time-scaled tree of the 227 A(H1N1)pdm09 HA gene sequences collected during the two A(H1N1)pdm09 epidemics showed that the isolates sampled in the pandemic season were mainly interspersed at the root of the tree, with only two significant clades respectively including 24 (clade E, pp = 0.93) and six isolates (clade F, pp = 0.97) sampled between August 2009 and January 2010. These two clades were also present in the global tree, but included multiple isolates from different countries in the world (Figure 1). On the contrary, most of the isolates collected in the post-pandemic season grouped into four highly significant clades (A–D) and a number of sub-clades, largely corresponding to those identified in the global tree. Clade A (pp = 1) included 70 strains isolated between December 2010 and March 2011; clade B (pp = 0.99) 31 isolates obtained between January and February 2011; clade C (pp = 0.88) 23 isolates obtained between November 2010 and January 2011; and clade D (pp = 0.99) 12 isolates sampled between January and March 2011 (Figure 2).

The time of the most recent common ancestor (tMRCA) of the Italian strains (corresponding to the tree root) was estimated to be a mean 25.3 months before March 2011 (i.e. February 2009), with a credibility interval of between 29 and 22 months (October 2008 and May 2009). The tMRCAs and the most probable months in the calendar time scale of the main clades and sub-clades estimated using the strict molecular clock model are shown in Table 1. The MRCA of the clustering strains circulating in the pandemic season (clades E–F) probably dated back to June 2009 (credibility interval between May and July 2009), whereas the common ancestors of the post-pandemic season clades (A–D) were dated between March and May 2010. The tMRCA of the internal sub-clades, which corresponds to the radiation of the circulating strains, varied between July and October 2010 (Table 1).

**Table 1:** Mean tMRCA estimates with credibility intervals (95%HPD) and the corresponding months of the tree root and main H1N1 clades and sub-clades.

Node	Months^1^	LHPD^2^	UHPD^3^	Date	Lower	Upper
Root	25.3	22.3	29.15	feb-09	may-09	oct-08
A	10.2	7.05	13.92	may-10	aug-10	jan-10
A′	6.32	4.3	8.5	sep-10	nov-10	jul-10
A′′	8.1	4.4	11.5	jul-10	nov-10	mar-10
B	11.9	7.68	16.01	mar-10	jul-10	nov-09
B′	7.1	4.3	10.4	aug-10	nov-10	may-10
B′′	5.3	3.6	8.1	oct-10	nov-10	jul-10
C	12.3	8.04	16.21	mar-10	jul-10	nov-09
C′	6.3	4.3	9.1	sep-10	nov-10	jun-10
C′′	6.7	3.9	10	aug-10	nov-10	may-10
D	12.2	8.02	16.27	mar-10	jul-10	nov-09
D′	4.6	2.4	6.9	oct-10	dec-10	aug-10
D′′	4.9	3.5	6.7	oct-10	dec-10	aug-10
E	21.1	20.32	22.14	jun-09	jul-09	may-09
F	21.2	21	21.59	jun-09	jun-09	may-09

1 Months before March 2011.

2 Lower 95% Highest Posterior Density.

3 Upper 95% Highest Posterior Density.

### Signature amino acid substitutions in the Italian clades and HA selection pressure analysis

The mean genetic distance was 0.3% (substitutions per 100 sites) (±0.1%) within the group of isolates obtained in the pandemic season, and 1% (±0.2%) within the group of isolates obtained during the post-pandemic season. The mean distance between the isolates of the two seasons was 0.9% (±0.2%).

A total of 16 codons showed mutations affecting more than 30% of the isolates included in at least one clade. Table 2 shows the clade-specific frequency of amino acid modifications at each site. Some codons were mutated in 100% of the isolates belonging to a specific clade: codons 205, 216 and 249 in clade A, 185 in clade B, 125 in clade C, 134 and 183 in clade D, 222 in clade E, and 32 in clade F. The most frequent mutation in our isolates was a D to N (or less frequently to K or H) substitution at codon 97 affecting the majority of clade A (98.6%) and B strains of the post-pandemic season (81.6%). Interestingly, codon 222 was mutated in 100% of the clade E strains (substitutions from D to E in all cases but one, in which the mutant amino acid was G) and was also sporadically mutated in 11 isolates dispersed in different clades although, in these cases, the mutant amino acid was always different from E.

**Table 2:** Clade-specific frequency of the most represented amino acid substitutions in HA protein.

Codon	%A	%B	%C	%D	%E	%F	%No clade	%Total
32	0	0	0	0	0	100	1,8	3,1
94	0	0	34,8	0	0	0	0	3,5
97	98,6	81,6		0	0	0	0	44,3
125	0	0	100	0	0	0	0	10,1
134	0	0	0	100	0	0	0	5,3
138	94,4	0	0	0	0	0	0	29,4
141	0	13,2	0	83,3	0	0	0	6,6
172	0	0	34,8	0	0	0	0	3,5
183	0	0	0	100	0	0	0	5,3
185	0	100	0	0	0	0	3,6	17,5
205	100	2,6	0	0	0	0	0	31,6
216	100	5,3	0	0	0	0	0	32
222	2,8	10,5	4,3	16,7	100	0	0	14,5
249	100	0	0	0	0	0	0	31,1
295	0	0	0	83,3	12,5	0	0	5,7
297	0	0	0	0	62,5	0	0	6,6

The ML estimate of the d_N_/d_S_ ratio (ω) gave a mean value of 0.43 (95% CI: 0.35–0.52), with no significant difference between different lineages (LRT from a global and local model 2Δlikelihood = 161.7, p>0.1). Using the Nielsen-Yang approach, we found that a model of evolution assuming site-specific selection fitted our data better than a neutral evolution model (M1-2Δlikelihood  = 13.06, p = 0.001 by LRT), and analysis of site-by-site selection pressure revealed that most of the methods used supported two sites under positive selection at a 90% level of significance (Table 3): codons 97 (from D to N/K/H) and 222 (from D to E/G/Y/N).

**Table 3:** Positive selected sites in the HA gene of influenza A(H1N1)pdm09 virus.

	Positive selection site data				Clade
Methods	Location	Position	Normalised d_N_/d_S_	P value	Specificity
SLAC^1^	RBS^2^ and AS^3^ (Ca2)	222	3,95	0,086	
FEL^4^		97	5,54	0,1	
	RBS and AS (Ca2)	222	9,94	0,037	
REL^5^		97	0,99		
		173	0,92		
	RBS and AS (Ca2)	222	1		
IFEL^6^		97	17,9	0,017	
	AS (Sa)	125	9,3	0,08	B2
	AS(Ca2)	141	8,8	0,07	
	AS (Sb)	185	7,8	0,09	B1
		249	4,9	0,1	A1
		297	5,1	0,1	

1 RBS: receptor binding site;

2 AS: antigenic site;

3 SLAC: single likelihood ancestor counting;

4 FEL; fixed effects likelihood;

5 REL, random effects likelihood;

6 IFEL; internal fixed effects likelihood.

Six sites were selected along the internal branches (Table 3), mainly corresponding to previously described clade-specific substitutions (codons 97, 125, 141, 185, 249 and 297). Interestingly, codon 222 was not one of the sites selected at population level.

### Population dynamics

Bayes factor comparison of four simple parametric (constant population size, exponential, expansion and logistic growth) and one piecewise demographic model (BSP) showed that the last fitted the data better than the others (Table S2).

**Figure 3:**
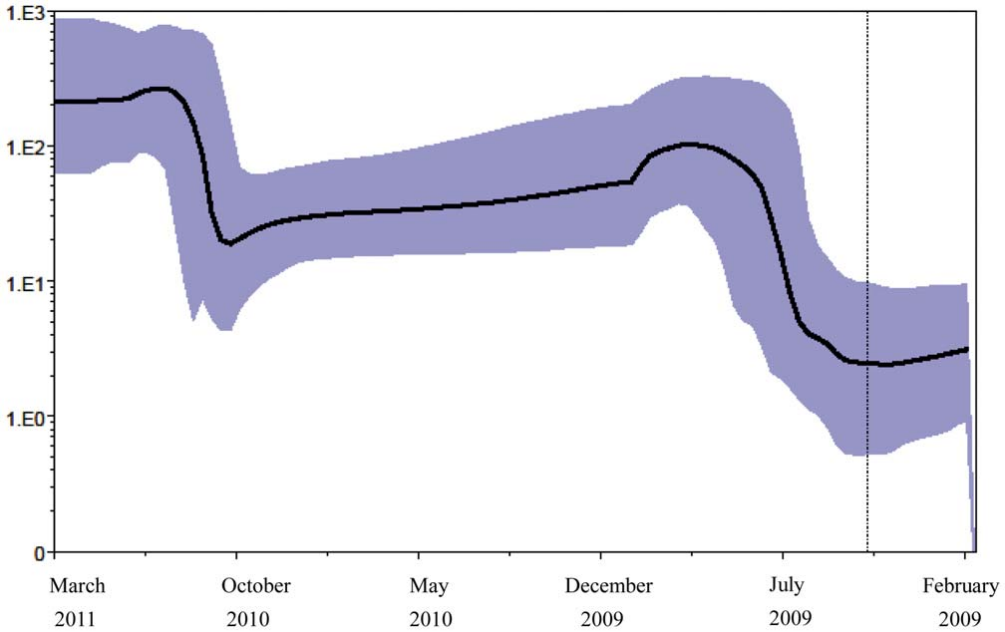
Bayesian skyline plot (with 20 coalescent interval groups) of the 227 Italian influenza A(H1N1)pdm09 sequences. Ordinate: the number of effective infections at time t (Ne(t)); abscissa: calendar months between the mean tMRCA estimate of the tree root and the most recent samples (March 2011). The thick solid line represents the median value, and the grey area the 95% HPD of the Ne(t) estimates. The vertical line indicates the 95% lower HPD tMRCA estimate of the tree root.

Analysis of the BSP (Figure 3) showed that the growth of the effective number of infections was biphasic: exponential growth started about 22–21 months before (corresponding to May–June 2009) and reached a plateau in December 2009; the second expansion of the epidemic started about in October 2010 and reached a plateau in December 2010. The estimation of the basic reproduction number (R_0_) on isolates sampled during the pandemic period, for a mean generation time of 2.8 days, varying between 1 and 5 days, gave a median value of 1.1, possibly changing between 1.0 and 1.3. The doubling time of the epidemic during pandemic period was estimated 28.11 days (CI 8.9–59.4 days).

### Local phylogeography

The local phylogeographical analysis was made by grouping the isolates on the basis of their sampling locations and building a spatial scaled phylogeny using the Bayesian framework. The location-annotated tree is shown in Figure S1.

**Figure 4:**
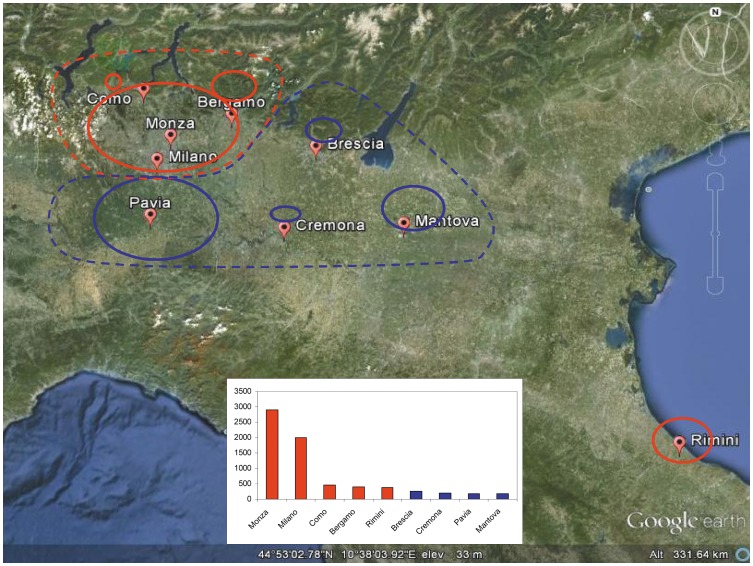
Map representing the localities of origin of the pandemic (red) and post-pandemic (blu) clades. The circle diameter is proportional to the number of isolates sampled in the locality. The low panel histogram represents the population density (number of inhabitants/km^2^).

Analysis showed that the first season of the epidemic was characterised by ancestors localised in Milan (the most probable location of the tree root) and in the area north of the city: the MRCA of clade E was most probably located in Milan (pp = 0.76), but the isolates included were from different places in Lombardy, whereas clade F was more restricted to Milan city and the hinterland. The post-pandemic season showed a more dispersed origin of the clades, which were localised in both northern and southern Lombardy: clades A and D most probably originated in the northern area of Milan (pp = 0.37), whereas clades B and C had MRCAs most probably located in southern Lombardy (pp = 0.37 and 0.34). The isolates included in all of the clades were dispersed throughout the region, including the southern part. The first wave of infection originated in localities with a high population density (≥382 inhabitants/km^2^), whereas the second wave involved less densely populated places (≤250 inhabitants/km^2^) (Figure 4).

## Discussion

We used a sophisticated Bayesian evolutionary framework for the molecular characterisation and reconstruction of the phylodynamics of the A(H1N1)09pdm influenza virus in northern Italy during two epidemics: the pandemic between summer and autumn 2009, and the post-pandemic period between November 2010 and March 2011.

In order to describe the Italian epidemics in the setting of the widespread diffusion of infection throughout the world, we analysed a total of 227 newly characterised northern Italian strains and a series of reference isolates from different countries retrieved from public databases. The first analysis included all of the patients' and reference isolates (global tree), and the second only the Italian strains, using a time-scaled phylogeny that assumed a strict molecular clock model.

Analysis of the trees suggested that the spread of the virus in Italy was the result of multiple independent introductions from different geographical areas because the Italian isolates sampled during the pandemic season were interspersed with sequences sampled in other countries at the root of the tree, and did not form any significant pure Italian clusters. The only exceptions were two clades in the Italian tree (E and F) that tended to group together in the global tree and included a number of isolates from other countries. Clade E was characterised by the presence of the signature substitution D222E, which has been previously described as circulating in the UK between July and September 2009 [23], thus suggesting possible importation from this country.

On the contrary, four or five highly significant pure Italian clades connected to the tree with long branches were observed during the post-pandemic season. Clade C was split into two different highly significant groups in the global tree, and clades A and D included a single non-Italian strain (one Turkish and one Tunisian).

These data suggest that multiple initial introductions of A(H1N1)09pdm in 2009 were followed by founder effects causing the local amplification of the infection in the post-pandemic season. In line with this hypothesis, the isolates of the post-pandemic wave showed greater intra-seasonal genetic divergence from those of the pandemic period, probably because of the typical effect of genetic drift on genetic variability, which tends to be less within groups but greater between groups.

In order to reconstruct the population dynamics of the H1N1 pandemic in Italy on a calendar time scale, we estimated the evolutionary rate of the Italian isolates using a better fitting strict molecular clock implemented in the Bayesian framework. Our estimates ranged from 3.5×10^−3^ to 6.4×10^−3^ substitutions/site/year, and were in line with those recently estimated for the HA gene by other authors [3], [24].

The tMRCA estimate of the tree root dating back to February 2009 is in line with the majority of the previous estimations, which place the origin of the pandemic H1N1 strain in January 2009, with intervals of credibility between late 2008 and March 2009 [3], [25], [26]. This pre-dates the first Italian identification of A(H1N1)09pdm infections and suggests that, also in Italy, unidentified infections in returning travellers may have occurred before the epidemiological alert [23]. The first laboratory-confirmed case in Lombardy was detected on 2 May 2009 in a traveller returning from Mexico, and was followed by further cases of travellers returning from Mexico or the USA. These virus introductions to Italy gave rise to a limited number of secondary and tertiary cases in chains of transmission and clusters in June and July 2009. A number of cases were detected in July in people travelling from the UK, where the first pandemic wave was ongoing. In line with this, the tMRCA of Italian clade E, which carried the mutation described in a large majority of the UK isolates, was estimated entering in Italy between May and July 2009. Following a decline in A(H1N1)pdm09 infections over the summer, the pandemic began spreading in mid-September 2009 at the time of the opening of schools. After an initial period characterised by a slow exponential increase, there was a sudden and sharp increase in community cases by mid-October that led to sustained community transmission and the pandemic wave, which peaked in mid-November and subsided in December 2009. The A(H1N1)pdm09 virus emerged again in November 2010 and played a major role as the main influenza virus circulating during the 2010–2011 influenza season. However, it is worth noting that the circulation of A(H1N1)pdm09 was rare during the last influenza season (2011–2012), when the A(H3N2) subtype returned to be predominant (European Centre for Disease Prevention and Control. Risk assessment. Seasonal influenza 2011–2012 in Europe. 9 March 2012. Available at: http://ecdc.europa.eu/en/publications/Publications/120312-TER-Seasonal-influenza-risk-assessment.pdf).

In line with these epidemiological data, the tMRCAs of the four specific Italian clades were dated spring 2010 (between March and May), and the radiation of the Italian strains (corresponding to the tMRCAs of the nodes inside the clades) were dated late summer and autumn 2010. Moreover, coalescent-based population dynamics revealed two phases in the exponential growth of the effective number of infections corresponding to the two seasons. The first exponential growth phase was between May and December 2009, and the second was between October 2010 and January 2011. The estimation of the basic reproductive number of the first pandemic period, gave a R_0_ close to the lower confidence limit estimated by Fraser, confirming the limited potential to spread of A(H1N1)pdm09, in comparison with previous pandemics [25].

In order to reconstruct the geographic dispersion of the infections, the northern Italian isolates were grouped on the basis of their place of isolation, and an estimate was made of the genetic flows between the different geographical areas. The analysis showed that the isolates obtained during the first pandemic season most probably originated in areas with high population densities, such as Milan and its north-western hinterland where there are important international airports, whereas the isolates of the second season were more dispersed and most probably originated in smaller and less densely populated areas such as southern Lombardy. Given the characteristics of the urbanisation of this area, these localities may represent the most probable geographical areas, in which the founder effect occurred, a hypothesis that is supported by the phylogeographical tree. This suggests that the geographical dispersion of A(H1N1)pdm09 was characterised by possibly gravity-like dynamics in which larger cities act as attractors and drive the spread of infection to their smaller counterparts [27]–[29].

The implementation of innovative advanced phylogenetic analysis methodologies to the study of emerging viruses at a molecular level will be of fundamental importance to improve concretely the epidemiological surveillance of emerging and re-emerging infections. Our present data shed light on the relationships between the evolutionary and phylogenetic characteristics of influenza A(H1N1)2009 virus and its geo-epidemiology, allowing to estimate essential parameters such as the transmission potential of the virus or the most probable path of geographical dispersion. Indeed, the opportunity of studying the emerging pathogens and analyzing their ecology, diffusion and evolution will allow generating an early response in case of outbreaks, particularly in a pandemic caused by viral pathogens able to spread quickly in the human population. The rapidity with which the history of the origin and dispersion of A(H1N1)pdm09 has been reconstructed on the basis of the available genomes [3], [25] it is an example of the power of the “phylodynamic” approach in the Public Health.

We also investigated the positive selection pressures acting on HA protein. Various algorithms revealed evidence of positive selection in the terminal and internal branches of six codons, most of which were fixed in the sequences of viruses belonging to different clades. The codons in the influenza HA gene identified as being positively selected are presumably encoding amino acid replacements that allow the virus to evade existing population immunity. In particular, two positions were observed with over 90% of significance. The D97N change was previously observed in influenza strains recovered from patients with fatal cases circulated in England [30] and in Indian isolates in co-occurrence with mutation E374K [31]. As regard the 222 residue, our data are in agreement with previous publications that report a positive selection on this site [30], [32], [33].

Particular attention was given to positions 187 and 222, which have recently been extensively studied because of their importance in receptor binding preference and cross-specific shifts [34]–[38]. In addition, the substitutions D222G/N/Y have been associated with severe influenza infection [36], [38]–[41], probably because of preferential binding to the α2–3 sialic acid receptor in lung tissue rather than to the α2–6 sialic acid receptor in the upper airways. The majority of A(H1N1)pdm09 viruses circulating during the pandemic did not bear the D222G/N/Y mutation, whereas the E change was present in all of the strains in clade E and was not associated with an increased risk of severe respiratory distress. However, it is worth noting that D222G/N/Y changes were observed in sequences belonging to all of the clades and that this position was under positive selection only in the terminal branches. The mutations at codon 222 associated with increased virulence were therefore not fixed in the population but seem to have emerged as a result of intra-patient selection. The segregation of these mutants in the lower respiratory tract and their lower affinity with sialic acid receptor (α2–6) are possible reasons for the unsuccessful spread of this virus.

In conclusion, on the basis of all of these observations, we can hypothesise that the A(H1N1)pdm09 virus was introduced into Italy as a result of multiple importations by travellers coming from affected foreign areas. The initial transmission networks originated in the more densely populated locations in northern Italy between summer and autumn 2009, after which repeated founder effects occurred in more dispersed populations living in smaller cities and originated new specifically Italian clades that characterised the second season of the pandemic between November 2010 and March 2011. This suggests a possible gravity-like model of phylogeographical spread.

## Supporting Information

Figure S1 **Bayesian phylogeographical tree with branches coloured on the basis of the most probable location.** The isolates were assigned to 14 different localities: MI =  Milan, MB =  Monza, BG =  Bergamo, BS =  Brescia, CR =  Cremona, VA =  Varese, CO =  Como, LC =  Lecco, MN =  Mantua, PV =  Pavia, PR =  Parma, RN =  Rimini, SV =  Savona, PA =  Palermo. The correspondences between the locations and colours are shown in the panel (bottom left), and the MRCA location posterior probabilities are indicated on the internal nodes of the tree.
(TIF)

Table S1 **A(H1N1)pdm09 HA sequences included in the dataset: characteristics of patients.**
(DOC)

Table S2 **Comparison of demographic models (H_0_: null hypothesis; H_A_: alternative hypothesis) by Bayes factor.**
(DOC)

## References

[1] Galiano M, Agapow PM, Thompson C, Platt S, Underwood A, et al (2011) Evolutionary pathways of the pandemic influenza A (H1N1) 2009 in the UK. PLoS One 6: e23779. 21887318 10.1371/journal.pone.0023779 PMC3161082

[2] Garten RJ, Davis CT, Russell CA, Shu B, Lindstrom S, et al (2009) Antigenic and genetic characteristics of swine-origin 2009 A(H1N1) influenza viruses circulating in humans. Science 325: 197–201. 19465683 10.1126/science.1176225 PMC3250984

[3] Smith GJ, Vijaykrishna D, Bahl J, Lycett SJ, Worobey M, et al (2009) Origins and evolutionary genomics of the 2009 swine-origin H1N1 influenza A epidemic. Nature 459: 1122–1125. 19516283 10.1038/nature08182

[4] Nelson MI, Tan Y, Ghedin E, Wentworth DE, St George K, et al (2010) Phylogeography of the spring and fall waves of the H1N1/09 pandemic influenza virus in the United States. J Virol 85: 828–834. 21068250 10.1128/JVI.01762-10 PMC3020026

[5] Network WGIS (2011) Manual for the laboratory diagnosis and virological surveillance of influenza. Geneva, Switzerland.

[6] Ellis JS, Chakraverty P, Clewley JP (1995) Genetic and antigenic variation in the haemagglutinin of recently circulating human influenza A (H3N2) viruses in the United Kingdom. Arch Virol 140: 1889–1904. 7503689 10.1007/BF01322680

[7] Posada D (2008) jModelTest: phylogenetic model averaging. Mol Biol Evol 25: 1253–1256. 18397919 10.1093/molbev/msn083

[8] Hasegawa M, Kishino H, Yano T (1985) Dating of the human-ape splitting by a molecular clock of mitochondrial DNA. J Mol Evol 22: 160–174. 3934395 10.1007/BF02101694

[9] Drummond AJ, Rambaut A (2007) BEAST: Bayesian evolutionary analysis by sampling trees. BMC Evol Biol 7: 214. 17996036 10.1186/1471-2148-7-214 PMC2247476

[10] Drummond AJ, Rambaut A, Shapiro B, Pybus OG (2005) Bayesian coalescent inference of past population dynamics from molecular sequences. Mol Biol Evol 22: 1185–1192. 15703244 10.1093/molbev/msi103

[11] Suchard MA, Weiss RE, Sinsheimer JS (2001) Bayesian selection of continuous-time Markov chain evolutionary models. Mol Biol Evol 18: 1001–1013. 11371589 10.1093/oxfordjournals.molbev.a003872

[12] Kass RE, Raftery AE (1995) Bayes factors. Journal of American Statistical Association 90: 773–795.

[13] Pybus OG, Charleston MA, Gupta S, Rambaut A, Holmes EC, et al (2001) The epidemic behavior of the hepatitis C virus. Science 292: 2323–2325. 11423661 10.1126/science.1058321

[14] Ferguson NM, Cummings DA, Cauchemez S, Fraser C, Riley S, et al (2005) Strategies for containing an emerging influenza pandemic in Southeast Asia. Nature 437: 209–214. 16079797 10.1038/nature04017

[15] Wallinga J, Lipsitch M (2007) How generation intervals shape the relationship between growth rates and reproductive numbers. Proc Biol Sci 274: 599–604. 17476782 10.1098/rspb.2006.3754 PMC1766383

[16] Walker PR, Pybus OG, Rambaut A, Holmes EC (2005) Comparative population dynamics of HIV-1 subtypes B and C: subtype-specific differences in patterns of epidemic growth. Infect Genet Evol 5: 199–208. 15737910 10.1016/j.meegid.2004.06.011

[17] Lemey P, Rambaut A, Drummond AJ, Suchard MA (2009) Bayesian phylogeography finds its roots. PLoS Comput Biol 5: e1000520. 19779555 10.1371/journal.pcbi.1000520 PMC2740835

[18] Kosakovsky Pond SL, Frost SD (2005) Not so different after all: a comparison of methods for detecting amino acid sites under selection. Mol Biol Evol 22: 1208–1222. 15703242 10.1093/molbev/msi105

[19] Suzuki Y, Gojobori T (1999) A method for detecting positive selection at single amino acid sites. Mol Biol Evol 16: 1315–1328. 10563013 10.1093/oxfordjournals.molbev.a026042

[20] Yang Z, Nielsen R (2000) Estimating synonymous and nonsynonymous substitution rates under realistic evolutionary models. Mol Biol Evol 17: 32–43. 10666704 10.1093/oxfordjournals.molbev.a026236

[21] Kosakovsky Pond SL, Frost SDW, Grossman Z, Gravenor MB, Richman DD, et al (2006) Adaptation to Different Human Populations by HIV-1 Revealed by Codon-Based Analyses. PLoS Comput Biol 2: e62. 16789820 10.1371/journal.pcbi.0020062 PMC1480537

[22] Pond SL, Frost SD, Muse SV (2005) HyPhy: hypothesis testing using phylogenies. Bioinformatics 21: 676–679. 15509596 10.1093/bioinformatics/bti079

[23] Baillie GJ, Galiano M, Agapow PM, Myers R, Chiam R, et al (2012) Evolutionary dynamics of local pandemic H1N1/2009 influenza virus lineages revealed by whole-genome analysis. J Virol 86: 11–18. 22013031 10.1128/JVI.05347-11 PMC3255882

[24] Rambaut A, Pybus OG, Nelson MI, Viboud C, Taubenberger JK, et al (2008) The genomic and epidemiological dynamics of human influenza A virus. Nature 453: 615–619. 18418375 10.1038/nature06945 PMC2441973

[25] Fraser C, Donnelly CA, Cauchemez S, Hanage WP, Van Kerkhove MD, et al (2009) Pandemic potential of a strain of influenza A (H1N1): early findings. Science 324: 1557–1561. 19433588 10.1126/science.1176062 PMC3735127

[26] Shiino T, Okabe N, Yasui Y, Sunagawa T, Ujike M, et al (2010) Molecular evolutionary analysis of the influenza A(H1N1)pdm, May-September, 2009: temporal and spatial spreading profile of the viruses in Japan. PLoS One 5: e11057. 20548780 10.1371/journal.pone.0011057 PMC2883557

[27] Holmes EC (2008) Evolutionary history and phylogeography of human viruses. Annu Rev Microbiol 62: 307–328. 18785840 10.1146/annurev.micro.62.081307.162912

[28] Li X, Tian H, Lai D, Zhang Z (2011) Validation of the gravity model in predicting the global spread of influenza. Int J Environ Res Public Health 8: 3134–3143. 21909295 10.3390/ijerph8083134 PMC3166731

[29] Lycett S, McLeish NJ, Robertson C, Carman W, Baillie G, et al (2012) Origin and Fate of A/H1N1 Influenza in Scotland during 2009. J Gen Virol. 93: 1253–1260. 10.1099/vir.0.039370-0 PMC3755513 22337637

[30] Mullick J, Cherian SS, Potdar VA, Chadha MS, Mishra AC (2011) Evolutionary dynamics of the influenza A pandemic (H1N1) 2009 virus with emphasis on Indian isolates: evidence for adaptive evolution in the HA gene. Infect Genet Evol 11: 997–1005. 21457796 10.1016/j.meegid.2011.03.015

[31] Ellis J, Galiano M, Pebody R, Lackenby A, Thompson C, et al (2011) Virological analysis of fatal influenza cases in the United Kingdom during the early wave of influenza in winter 2010/11. Euro Surveill 16: 19760. 21223836

[32] Li W, Shi W, Qiao H, Ho SY, Luo A, et al (2011) Positive selection on hemagglutinin and neuraminidase genes of H1N1 influenza viruses. Virol J 8: 183. 21507270 10.1186/1743-422X-8-183 PMC3094300

[33] Tse H, Kao RY, Wu WL, Lim WW, Chen H, et al (2011) Structural basis and sequence co-evolution analysis of the hemagglutinin protein of pandemic influenza A/H1N1 (2009) virus. Exp Biol Med (Maywood) 236: 915–925. 21727184 10.1258/ebm.2011.010264

[34] Glaser L, Stevens J, Zamarin D, Wilson IA, Garcia-Sastre A, et al (2005) A single amino acid substitution in 1918 influenza virus hemagglutinin changes receptor binding specificity. J Virol 79: 11533–11536. 16103207 10.1128/JVI.79.17.11533-11536.2005 PMC1193621

[35] Hu W (2010) Quantifying the effects of mutations on receptor binding specificity of influenza viruses. J Biomedical Science and Engineering 3: 227–240.

[36] Liu Y, Childs RA, Matrosovich T, Wharton S, Palma AS, et al (2010) Altered receptor specificity and cell tropism of D222G hemagglutinin mutants isolated from fatal cases of pandemic A(H1N1) 2009 influenza virus. J Virol 84: 12069–12074. 20826688 10.1128/JVI.01639-10 PMC2977873

[37] Shen J, Ma J, Wang Q (2009) Evolutionary trends of A(H1N1) influenza virus hemagglutinin since 1918. PLoS One 4: e7789. 19924230 10.1371/journal.pone.0007789 PMC2773012

[38] Rogers GN, D'Souza BL (1989) Receptor binding properties of human and animal H1 influenza virus isolates. Virology 173: 317–322. 2815586 10.1016/0042-6822(89)90249-3

[39] Baldanti F, Campanini G, Piralla A, Rovida F, Braschi A, et al (2011) Severe outcome of influenza A/H1N1/09v infection associated with 222G/N polymorphisms in the haemagglutinin: a multicentre study. Clin Microbiol Infect 17: 1166–1169. 20946414 10.1111/j.1469-0691.2010.03403.x

[40] Kilander A, Rykkvin R, Dudman SG, Hungnes O (2010) Observed association between the HA1 mutation D222G in the 2009 pandemic influenza A(H1N1) virus and severe clinical outcome, Norway 2009–2010. Euro Surveill 15. 10.2807/ese.15.09.19498-en 20214869

[41] Piralla A, Pariani E, Rovida F, Campanini G, Muzzi A, et al (2011) Segregation of virulent influenza A(H1N1) variants in the lower respiratory tract of critically ill patients during the 2010–2011 seasonal epidemic. PLoS One 6: e28332. 22194826 10.1371/journal.pone.0028332 PMC3237448

