# Supplementary figures and images for: Reconstruction of the Evolutionary Dynamics of the A(H1N1)pdm09 Influenza Virus in Italy during the Pandemic and Post-Pandemic Phases
Source: PLoS One. 2012 Nov 9;7(11):e47517. doi: 10.1371/journal.pone.0047517 (PMC3494699; doi:10.1371/journal.pone.0047517)

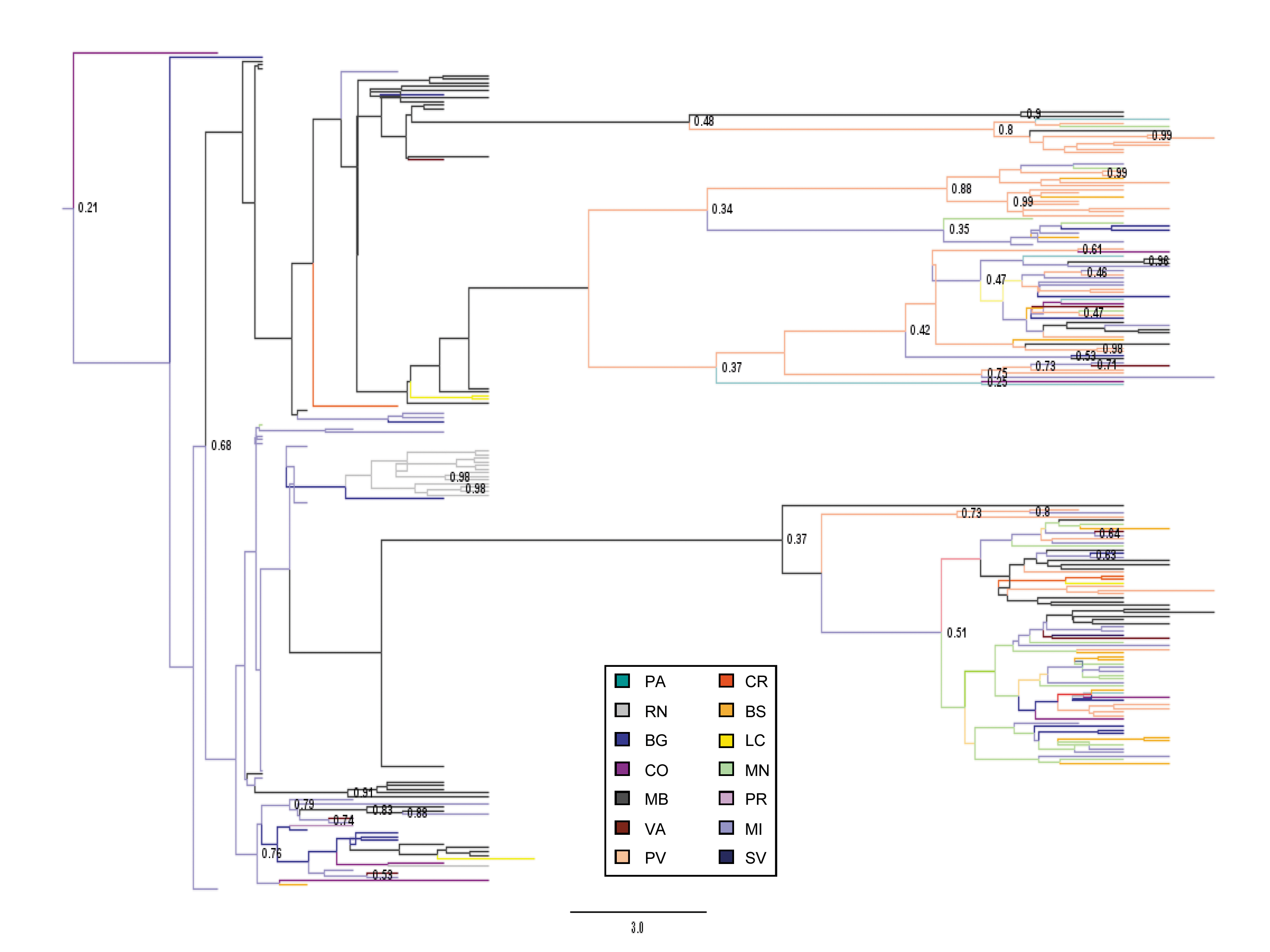

Supplement: Figure S1 — Bayesian phylogeographical tree with branches coloured on the basis of the most probable location. The isolates were assigned to 14 different localities: MI = Milan, MB = Monza, BG = Bergamo, BS = Brescia, CR = Cremona, VA = Varese, CO = Como, LC = Lecco, MN = Mantua, PV = Pavia, PR = Parma, RN = Rimini, SV = Savona, PA = Palermo. The correspondences between the locations and colours are shown in the panel (bottom left), and the MRCA location posterior probabilities are indicated on the internal nodes of the tree. (TIF) [file pone.0047517.s001.tif]
