# Supplementary material for: Reconstruction of the Evolutionary Dynamics of the A(H1N1)pdm09 Influenza Virus in Italy during the Pandemic and Post-Pandemic Phases
Source: PLoS One. 2012 Nov 9;7(11):e47517. doi: 10.1371/journal.pone.0047517 (PMC3494699; doi:10.1371/journal.pone.0047517)
Supplement: Table S1 — A(H1N1)pdm09 HA sequences included in the dataset: characteristics of patients. (DOC) [file pone.0047517.s002.doc]

**Table S1**. A(H1N1)pdm09 HA sequences included in the dataset: characteristics of patients.

|  | **Pandemic^a^** | **Post-pandemic^b^** | **Total** |
| --- | --- | --- | --- |
| # of specimens | 3875 | 1969 | 5844 |
| # of specimens with positive A(H1N1)pdm09 detection | 1275 | 388 | 1633 |
| A(H1N1)pdm09 HA sequences | 84 | 144 | 228 |
| Males/Females | 55/29 | 89/55 | 144/84 |
| Median age, years | 12 | 45 | 33 |
| (IQR^1^; range) | (22; 0.2-79) | (25; 1-85) | (38; 0.2-85) |
| # of subjects with URTI^2^ | 44 | 63 | 107 |
| # of subjects with LRTI^3^ | 35 | 40 | 75 |
| # of subjects with ARDS^4^ | 5 | 41 | 46 |

^a^May 2009-April 2010

^b^May 2010-April 2011

^1^IQR: Interquartile range.

^2^URTI: upper respiratory tract infection characterised by ILI symptoms: i.e. abrupt onset of fever (>38.0°C), one or more respiratory symptoms (non-productive cough, sore throat, rhinitis), and one or more systemic symptoms (myalgia, headache, severe malaise) not requiring hospitalisation, the administration of antiviral drugs or oxygen supplementation.

^3^LRTI: lower respiratory tract infection characterised by positive chest radiography and/or reduced pO_2_ blood saturation (<90%) requiring admission to units other than ICUs for antiviral and/or oxygen supplementation by means of non-invasive assisted ventilation procedures.

^4^ARDS: acute respiratory distress syndrome characterised by bilateral alveolar infiltrates on chest radiography and rapidly progressive hypoxemia, with admission to ICUs for invasive mechanical ventilation or ECMO procedures.
