# Supplementary material for: Reconstruction of the Evolutionary Dynamics of the A(H1N1)pdm09 Influenza Virus in Italy during the Pandemic and Post-Pandemic Phases
Source: PLoS One. 2012 Nov 9;7(11):e47517. doi: 10.1371/journal.pone.0047517 (PMC3494699; doi:10.1371/journal.pone.0047517)
Supplement: Table S2 — Comparison of demographic models (H0: null hypothesis; HA: alternative hypothesis) by Bayes factor. (DOC) [file pone.0047517.s003.doc]

**Table S2. Comparison of demographic models (H_0_: null hypothesis; H_A_: alternative hypothesis) by Bayes factor.**

| **H_0_** | **H_A_** | **2lnBF** | **Selected model** |
| --- | --- | --- | --- |
| Constant | Exponential | 58.6 | Exponential |
| Exponential | Expansion | -36 | Exponential |
| Exponential | Logistic | 3.8 | Exponential |
| Exponential | BSP | 76.56 | BSP |
